# Genetic Polymorphisms of the Main Transcription Factors for Adiponectin Gene Promoter in Regulation of Adiponectin Levels: Association Analysis in Three European Cohorts

**DOI:** 10.1371/journal.pone.0052497

**Published:** 2012-12-21

**Authors:** Lyudmyla Kedenko, Claudia Lamina, Tobias Kiesslich, Karen Kapur, Sven Bergmann, Dawn Waterworth, Iris M. Heid, H.-Erich Wichmann, Igor Kedenko, Florian Kronenberg, Bernhard Paulweber

**Affiliations:** 1 University Clinic for Internal Medicine I, Paracelsus Medical University Salzburg, Austria; 2 Division of Genetic Epidemiology, Innsbruck Medical University, Innsbruck, Austria; 3 Department of Medical Genetics, University of Lausanne, Switzerland; 4 Swiss Institute of Bioinformatics, Lausanne, Switzerland; 5 Genetics, GlaxoSmithKline, King of Prussia, Philadelphia, United States of America; 6 Department of Epidemiology and Preventive Medicine, Regensburg University Medical Center, Regensburg, Germany; 7 Institute of Epidemiology I, Helmholtz Center Munich, German Research Center for Environmental Health, Neuherberg, Germany; 8 Institute of Medical Informatics, Biometry and Epidemiology, Chair of Epidemiology, Ludwig-Maximilians-Universität, Munich, Germany; 9 Klinikum Grosshadern, Munich, Germany; University of Tampere, Finland

## Abstract

Adiponectin serum concentrations are an important biomarker in cardiovascular epidemiology with heritability etimates of 30–70%. However, known genetic variants in the adiponectin gene locus (*ADIPOQ*) account for only 2%–8% of its variance. As transcription factors are thought to play an under-acknowledged role in carrying functional variants, we hypothesized that genetic polymorphisms in genes coding for the main transcription factors for the *ADIPOQ* promoter influence adiponectin levels. Single nucleotide polymorphisms (SNPs) at these genes were selected based on the haplotype block structure and previously published evidence to be associated with adiponectin levels. We performed association analyses of the 24 selected SNPs at forkhead box O1 (*FOXO1)*, sterol-regulatory-element-binding transcription factor 1 (*SREBF1)*, sirtuin 1 (*SIRT1)*, peroxisome-proliferator-activated receptor gamma (*PPARG)* and transcription factor activating enhancer binding protein 2 beta (*TFAP2B)* gene loci with adiponectin levels in three different European cohorts: SAPHIR (n = 1742), KORA F3 (n = 1636) and CoLaus (n = 5355). In each study population, the association of SNPs with adiponectin levels on log-scale was tested using linear regression adjusted for age, sex and body mass index, applying both an additive and a recessive genetic model. A pooled effect size was obtained by meta-analysis assuming a fixed effects model. We applied a significance threshold of 0.0033 accounting for the multiple testing situation. A significant association was only found for variants within *SREBF1* applying an additive genetic model (smallest p-value for rs1889018 on log(adiponectin) = 0.002, β on original scale = −0.217 µg/ml), explaining ∼0.4% of variation of adiponectin levels. Recessive genetic models or haplotype analyses of the *FOXO1*, *SREBF1*, *SIRT1*, *TFAPB2B* genes or sex-stratified analyses did not reveal additional information on the regulation of adiponectin levels. The role of genetic variations at the *SREBF1* gene in regulating adiponectin needs further investigation by functional studies.

## Introduction

Adipose tissue secretes a number of peptides referred to as adipocytokines or adipokines [1]. One such adipocytokine is adiponectin encoded by *ADIPOQ* (also known as *APM1*) which is involved in regulation of insulin sensitivity, carbohydrate and lipid metabolism, immunological responses, and cardiovascular functions [2], [3]. Several studies indicated that 39%–70% of the variability in adiponectin levels is governed by genetic factors [4]–[7]. During the last decade, several polymorphisms at the *ADIPOQ* locus have been tested for association with adiponectin levels [8], [9]. Results of a meta-analysis and genome-wide association (GWA) studies indicated a role of variants in the *ADIPOQ* gene region in modulation of adiponectin levels [10], [11]. Although this locus explains exceptionally high 2%–8% of the adiponectin levels [8], [11], [12], a major fraction of heritability is still unexplained. The results of different GWA studies and linkage analyses suggested that different genomic regions are likely to be involved in regulation of adiponectin levels – either via a primary influence or through pathways influencing body composition [10], [11], [13]–[17].

The adiponectin gene promoter region contains binding sites for various types of nuclear receptors (PPARG2, LRH, RXR), transcription factors (CEBPA, SREBP1c, TFAP2B, FOXO1, SP1) and at least three co-regulators of transcription factors (SIRT1, NCOR1 and NCOR2) [18]–[20]. Kita et al. demonstrated that the adiponectin promoter region from −676 to +41 is sufficient for promoter activity and that the region from −676 to −416 is crucial for basal promoter activity [21]. This region consists of putative SREBP-responsive element (−676 to −416) and CEBP*-*responsive element (−416 to +41) which were both required for promoter activity. Furthermore, it was shown that FOXO1 up-regulates adiponectin gene transcription through a FOXO1-response element in the adiponectin promoter containing two adjacent FOXO1 binding sites [22]. SIRT1 increases adiponectin transcription in adipocytes by activating FOXO1 and enhancing FOXO1 and CEBPA interaction. Low expression of SIRT1 and FOXO1 can lead to impaired FOXO1-CEBPA complex formation, which might contribute to the diminished adiponectin expression in obesity [22]. PPARG2 may directly bind to the human adiponectin promoter by forming heterodimers with RXR and increase adiponectin promoter activity [18]. Moreover, promoter activity of the adiponectin gene is inhibited by over-expression of TFAP2B and enhanced by knockdown of its endogenous expression [23].

These lines of evidence indicate that several transcription factors and their co-regulators are involved in adiponectin gene expression: some through binding to the adiponectin promoter and increasing promoter activity, others through negative regulation of adiponectin gene expression. Taking this into consideration, we hypothesized that genetic polymorphisms in these gene regions may influence adiponectin gene transcription and adiponectin levels. To evaluate this hypothesis, we performed association analyses on the relation of selected polymorphisms in main adiponectin transcription factors for adiponectin promoter with the adiponectin levels in three different European cohorts.

## Materials and Methods

### Study Populations

#### SAPHIR Study

The SAPHIR study has been initiated in the year 1999 as a population-based prospective study that investigates genetic and environmental factors contributing to atherosclerotic vascular diseases [8]. Study participants were recruited by a health screening programs in large companies in and around the city of Salzburg, Austria. The study comprises 1770 healthy unrelated Caucasian subjects (663 females and 1107 males aged 39–67 years). At baseline, all participants were subjected to a comprehensive examination – detailed personal and family history, physical, instrumental and laboratory investigations. Serum adiponectin levels were measured by an enzyme-linked immunosorbent assay kit from BioCat (Heidelberg, Germany). DNA was available from 1760 participants and all relevant variables for analyses were available for 1742 participants.

#### KORA F3 Study

The KORA F3 Study is the 10 year follow-up of the third survey from the KORA-Study (Cooperative Health Research in the Region of Augsburg), a population-based sample from the general population of the South-German city of Augsburg and surrounding counties from 1994/1995. The KORA surveys have been described in detail previously [24]. Genome-wide genotype data were available for a subsample of 1644 individuals with all relevant variables available for 1636 individuals. Serum levels of adiponectin were measured by ELISA from Mercodia (Uppsala, Sweden).

#### CoLaus Study

The CoLaus study is a single-center, cross-sectional study which included 6188 Caucasian subjects aged 35 to 75 years from the city of Lausanne in Switzerland [25]. The major goal of the CoLaus study is investigation of prevalence, severity and molecular architecture of cardiovascular risk factors in a Lausanne population. Recruitment began in June 2003 and ended in May 2006. For 5435 participants, genome-wide genotype data are available. The current analysis included 5355 extensively phenotyped participants from this study. Plasma adiponectin levels were measured using the ELISA assay from R&D Systems (MN, USA).

### Ethics Statement

All clinical investigations were conducted according to the principles expressed in the Declaration of Helsinki. Participants from all 3 studies provided written informed consent and the studies were approved by the local ethical committees (SAPHIR Study, Ethical Committee of Land Salzburg; KORA F3, local ethical committee of Bayerische Landesärztekammer; CoLaus, Institutional Ethic’s Committee of the University of Lausanne).

### Selection of Transcription Factors

Three main transcription factors acting on the adiponectin promoter (FOXO1, SREBP1c, TFAP2B) were selected based on previously published data on their important role in regulation of human adiponectin promoter activity or association of genetic polymorphisms at these genes with adiponectin levels [21], [22], [26], [27]. CEBPA was excluded from our consideration because of controversial data about the CEBPA binding site at the adiponectin gene promoter or intron I [21], [28], [29] and lack of evidence for a role of genetic variation at this locus in control of adiponectin levels. One co-regulator of transcription factors (SIRT1) was added based on its interaction with FOXO1 in regulation of adiponectin expression [22].

### SNP Selection

Genotype data of the main transcription factors *(FOXO1, SREBF1, TFAP2B*) and one co-regulator of the transcription factors (*SIRT1*) for adiponectin promoter were downloaded from HapMap Data (Phase III/Rel.#2, Feb 09) (http://hapmap.ncbi.nlm.nih.gov/cgi-perl/gbrowse/hapmap3r2_B36/). Then data were transferred to SNP tagger (http://www.broad.mit.edu/mpg/tagger/server.html) to identify haplotype-tagging SNPs.

Using the haplotype block structure we selected a maximally informative subset of validated SNPs with minor allele frequency of ≥5% and pairwise r^2^≥80% (*FOXO1* MAF = 10%, r^2^ = 80%; *SREBF1* MAF = 5%, r^2^ = 100%; *TFAP2B* MAF = 5%, r^2^ = 80%; *SIRT1* MAF = 5%, r^2^ = 80%). Haplotype frequencies in each gene were estimated by implementation of the expectation maximization algorithm. Four SNPs (rs2236319, rs3740051 at *SIRT1,* rs987237 at *TFAP2B and* rs1801282 at *PPARG)* were included additionally based on the previous publications [30]–[33].

### Genotyping

Genomic DNA was isolated from whole blood in all three populations according to manufacturer’s protocols. Genotyping in SAPHIR cohort was performed using 5' nuclease allelic discrimination TaqMan genotyping method and pre-designed assays from Applied Biosystems (Foster City, CA, USA) according to the manufacturer’s instructions.

After the SNPs have been genotyped in SAPHIR, in-silico replication has been performed in the KORA F3 and CoLaus cohorts. For both populations, imputed genome-wide genotypes are available from which the respective SNPs were selected. Original genotyping for KORA F3 and CoLaus studies were performed using the Affymetrix GeneChip Human Mapping 500K Array Set (Affymetrix, Santa Clara, USA). Genotypes were determined using the BRLMM clustering algorithm (Bayesian Robust Linear Modeling using Mahalanobis distance, Affymetrix 500K Array Set) [34]. Imputation of genotypes was performed with the software MACH v1.0.9 [35] in KORA F3 and IMPUTE version 0.2.0 [36] in the CoLaus study.

### Statistical Analysis

In each study population, the association of each SNP with log-transformed adiponectin levels using linear regression models adjusted for age, sex and body mass index (BMI) applying an additive as well as recessive genetic model was analyzed. The analyses were also conducted stratified for sex, in this case only adjusting for age and BMI. A pooled effect size for all participants as well as for men and women separately was obtained by meta-analysis assuming a fixed effects model. The possibility of sex-specific effects has been tested on each SNP using a t-test based on the meta-analyzed effect estimates and standard errors on the original scale of adiponectin [37].

Correction for multiple testing was applied on independent number of tests for the main analyses (additive model). This number was calculated using the effective number of loci [38], which accounts for the correlation structure between the SNPs. The percentage of explained variance per SNP and pair-wise linkage disequilibrium (LD) between SNPs (D' and R^2^) was obtained using data from the SAPHIR study only. Also in SAPHIR, haplotypes were estimated for all genes except *PPARG* by the expectation maximization algorithm using the haplo.stats package http://CRAN.R-project.org/package=haplo.stats) in the R software environment [39]. Subsequent association analysis of the number of haplotype copies on log-transformed adiponectin was adjusted for age, sex and BMI.

## Results

### Patient and Genotype Characteristics

Clinical characteristics and adiponectin levels of the three study populations are presented in Table 1. All three study populations were comparable with respect to the phenotype studied and adjusting variables with the exception of the higher mean age and higher rate of type 2 diabetes (T2D) in the KORA F3 Study. There was a higher frequency of male participants in the SAPHIR population (62.7%) compared KORA F3 (49.5%) and CoLaus cohorts (47.3%).

**Table 1:** Clinical characteristics of the subjects from SAPHIR, KORA F3 and CoLaus studies (means ± SD or numbers (%)), for whom all relevant variables and genotypes are available.

Parameters	SAPHIR	KORA F3	CoLaus
Number	1742	1636	5355
Male individuals	1092 (62.7%)	809 (49.5%)	2531 (47.3%)
Age (years)	51.4±6.0	62.5±10.1	53.4±11.0
BMI (kg/m^2^)	26.8±4.1	28.1±4.5	25.8±4.5
Adiponectin (µg/ml)	8.58±4.58	10.61±4.65	10.05±8.00
Type 2 Diabetes (%)	58 (3.3%)	179 (10.9%)	348 (6.5%)

Initially, we genotyped 24 SNPs at *FOXO1*, *SREBF1*, *PPARG*, *TFAP2B* and *SIRT1* genes in SAPHIR cohort and added in-silico replication of the respective SNPs using the imputed genotypes from KORA F3 and CoLaus. Table 2 shows the corresponding descriptive statistics of the genotypes. The minor allele frequencies were comparable between all three populations. Issues regarding the genotyping quality could only be detected in four SNPs. In *TFAP2B* gene rs2143079 was poorly imputed (imputation quality score RSQR<60% in both in KORA F3 and CoLaus) and rs1569777 showed a deviation from the Hardy-Weinberg equilibrium in SAPHIR. Also in *FOXO1,* two SNPs (rs10507486 and rs17446614) had a deviation from the Hardy-Weinberg equilibrium in SAPHIR cohort (Table 2).

**Table 2:** Characteristics of the 24 SNPs in SAPHIR, KORA F3 and CoLaus, including genotype quality (call rate or imputation quality RSQR).

	Alleles	SAPHIR (n = 1760)				KORA F3 (n = 1644)				CoLaus (n = 5435)			
SNP	Minor/Major*	MAF	Genotype counts**	HWEp***	Call rate	MAF	Genotype counts**	HWEp***	RSQR	MAF	Genotype counts**	HWEp***	RSQR
***FOXO1***													
rs10507486	*A/G*	0.203	1135/529/91	0.006	0.997	0.216	1016/546/82	0.424	0.999	0.194	3543/1673/219	0.206	1.000
rs17446593	*G/A*	0.178	1186/513/55	1.000	0.997	0.177	1111/483/50	0.866	0.905	0.168	3781/1478/174	0.027	0.943
rs17446614	*A/G*	0.155	1259/430/55	0.017	0.991	0.167	1144/451/49	0.594	1.000	0.153	3916/1378/141	0.155	1.000
rs2297627	*G/A*	0.304	867/710/180	0.055	0.998	0.310	788/692/164	0.526	1.000	0.309	2616/2280/539	0.179	1.000
rs2721068	*C/T*	0.260	958/666/121	0.709	0.991	0.258	902/636/106	0.699	0.963	0.254	3061/1982/391	0.005	0.977
***PPARG***													
rs1801282	*G/C*	0.137	1309/413/35	0.688	0.998	0.143	1203/411/30	0.546	0.997	0.118	4248/1095/92	0.030	1.000
***SIRT1***													
rs10823108	*A/G*	0.063	1546/203/9	0.414	0.999	0.074	1410/224/10	0.718	0.998	0.065	4757/649/29	0.176	0.998
rs12413112	*A/G*	0.120	1365/363/29	0.367	0.998	0.138	1220/395/29	0.755	0.996	0.124	4174/1174/87	0.850	0.996
rs1467568	*A/G*	0.340	769/768/209	0.424	0.992	0.362	679/739/226	0.286	0.998	0.336	2390/2433/611	0.759	0.997
rs2236319	*G/A*	0.063	1543/207/7	1.000	0.998	0.074	1412/222/10	0.716	0.999	0.065	4759/647/29	0.141	0.997
rs2273773	*C/T*	0.063	1544/205/8	0.683	0.998	0.074	1410/224/10	0.718	0.943	0.067	4738/667/30	0.176	0.972
rs3740051	*G/A*	0.063	1546/204/8	0.682	0.999	0.073	1413/221/10	0.589	0.999	0.065	4759/646/29	0.141	0.997
***SREBF1***													
rs11656665	*G/A*	0.383	657/847/246	0.336	0.994	0.387	618/778/248	0.917	0.942	0.404	1939/2589/898	0.445	0.952
rs11868035	*A/G*	0.303	845/750/155	0.571	0.994	0.301	796/705/143	0.482	0.882	0.309	2582/2329/515	0.704	0.866
rs1889018	*G/A*	0.378	671/831/246	0.684	0.993	0.391	610/783/251	1.000	0.981	0.404	1948/2583/904	0.409	0.994
rs2236513	*A/C*	0.373	690/812/247	0.759	0.994	0.384	622/783/239	0.794	0.980	0.399	1987/2558/890	0.172	0.984
rs2297508	*C/G*	0.365	705/820/230	0.757	0.997	0.372	652/762/230	0.752	0.909	0.387	2054/2532/838	0.207	0.900
***TFAP2B***													
rs1569777	*T/C*	0.117	1406/278/66	0.000	0.994	0.109	1307/315/22	0.526	0.936	0.126	4145/1208/81	0.393	0.957
rs2076309	*T/C*	0.432	555/885/316	0.264	0.998	0.450	504/802/338	0.584	0.988	0.455	1609/2702/1124	0.867	0.988
rs2143079	*G/C*	0.052	1578/179/2	0.231	0.999	0.063	1440/202/2	0.087	0.540	0.054	4865/553/16	1.000	0.574
rs2206277	*T/C*	0.202	1117/563/73	0.825	0.996	0.165	1147/451/46	0.858	0.922	0.149	3928/1388/118	1.000	0.957
rs2857506	*A/G*	0.117	1338/358/22	0.816	0.976	0.113	1294/330/20	1.000	0.949	0.105	4350/1025/60	0.881	0.974
rs7771651	*T/A*	0.145	1280/441/34	0.631	0.997	0.150	1185/424/35	0.772	0.992	0.161	3811/1497/127	0.212	0.985
rs987237	*G/A*	0.199	1123/571/64	0.454	0.999	0.164	1150/449/45	0.857	0.929	0.149	3933/1384/117	1.000	0.961

* Minor and Major alleles based on the plus-strand.

** Number of homozygotes for the major allele/heterozygotes/homozygotes for the rare allele; For KORA F3 and CoLaus, where imputed genotype scores have been used, this are the numbers of the “best guess” genotypes (KORA F3) and rounded sum of genotype scores.

*** Based on exact test of Hardy-Weinberg Equilibrium (HWE).

### Association Analysis

**Figure 1:**
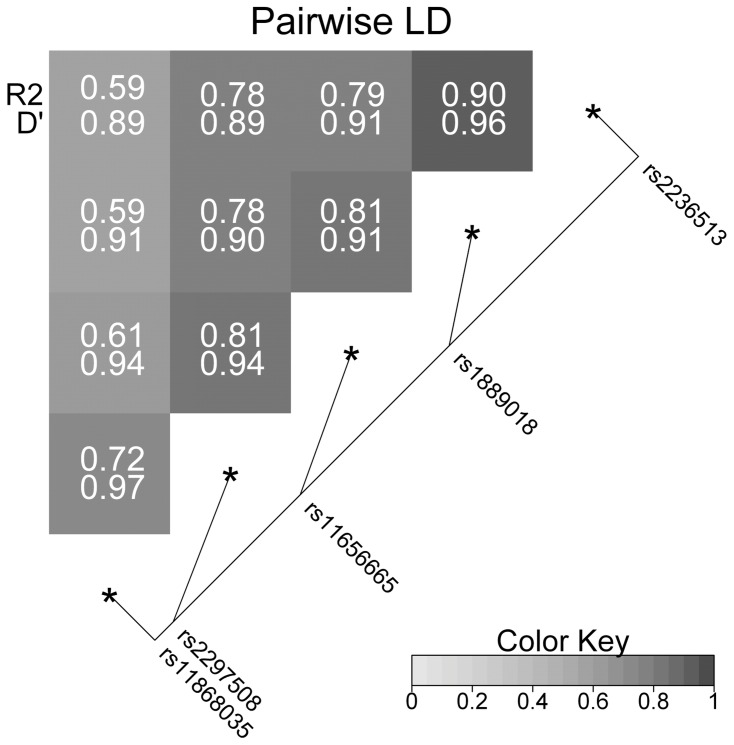
Linkage disequilibrium structure across the *SREBF1* single nucleotide polymorphisms. The pair wise linkage disequilibrium (R^2^ and D’) is given for each pair of single nucleotide polymorphisms. Color-coding is based on R^2^. The diagonal line indicates the physical position of the single nucleotide polymorphisms relative to each other.

We evaluated the association of genetic variants in the main transcription factors (*FOXO1*, *SREBF1*, *PPARG*, *TFAP2B*) and their co-regulator (*SIRT1*) for *ADIPOQ* promoter with adiponectin level. Meta-analysis of additive linear regression models adjusted for age, sex, and BMI revealed association with log(adiponectin) in all 5 selected SNPs in the *SREBF1* gene. After calculating the number of independent SNPs, which is 15 out of the 24 selected SNPs, and correcting for multiple testing (α = 0.05/15 = 0.0033) two SNPs from the *SREBF1* gene remained significant: rs1889018 (p = 0.002) and rs2236513 (p = 0.003). Table 3 shows the results for all three populations as well as the combined effects. For rs1889018, for example, each copy of the minor allele leads to a reduction of the adiponectin level of 0.217 µg/ml. This corresponds to an explained variance of ∼0.4% as calculated from the SAPHIR Study. All selected SNPs within *SREBF1* are highly correlated (Figure 1).

**Table 3:** Linear model results on the 24 selected SNPs in the SAPHIR, KORA F3 and CoLaus study using an additive genetic model, adjusted for age, sex and BMI, as well as the combined fixed effects meta-analysis results.

	SAPHIR (n = 1742)		KORA F3 (n = 1636)		CoLaus (n = 5355)		Meta-analysis results		Meta-analysis results, separated for menand women, t-test on difference		
SNP	ß (SE)	P	ß (SE)	P	ß (SE)	P	ß (SE)	P	ß (SE) men	ß (SE) women	P (diff)
***FOXO1***											
rs10507486	0.23 (0.164)	0.23	−0.169 (0.175)	0.62	−0.075 (0.18)	0.70	0.007 (0.1)	0.80	0.003 (0.107)	−0.007 (0.173)	0.96
rs17446593	−0.161 (0.178)	0.28	0.006 (0.191)	0.60	0.07 (0.195)	0.89	−0.036 (0.108)	0.98	−0.009 (0.116)	−0.047 (0.189)	0.84
rs17446614	−0.092 (0.183)	0.56	−0.123 (0.194)	0.87	−0.026 (0.197)	0.99	−0.081 (0.11)	0.90	0.031 (0.118)	−0.208 (0.193)	0.21
rs2297627	0.037 (0.144)	0.56	−0.136 (0.156)	0.61	−0.099 (0.154)	0.35	−0.06 (0.087)	0.40	−0.003 (0.094)	−0.129 (0.151)	0.40
rs2721068	−0.167 (0.154)	0.51	−0.046 (0.167)	0.82	−0.073 (0.163)	0.48	−0.099 (0.093)	0.46	0.009 (0.101)	−0.211 (0.161)	0.17
***PPARG***											
rs1801282	−0.197 (0.195)	0.25	0.135 (0.21)	0.33	0.103 (0.22)	0.87	0.001 (0.12)	0.89	−0.235 (0.129)	0.288 (0.208)	0.012
***SIRT1***											
rs10823108	−0.17 (0.277)	0.83	−0.155 (0.276)	0.38	0.453 (0.288)	0.12	0.032 (0.162)	0.30	−0.06 (0.169)	0.171 (0.291)	0.42
rs12413112	0.033 (0.206)	0.69	−0.051 (0.212)	0.81	0.083 (0.217)	0.56	0.021 (0.122)	0.82	−0.117 (0.13)	0.162 (0.216)	0.19
rs1467568	0.023 (0.142)	0.55	0.054 (0.149)	0.83	0.202 (0.152)	0.08	0.089 (0.085)	0.04	0.098 (0.091)	0.095 (0.149)	0.98
rs2236319	−0.168 (0.279)	0.94	−0.15 (0.277)	0.41	0.455 (0.288)	0.13	0.036 (0.162)	0.31	−0.045 (0.171)	0.161 (0.291)	0.47
rs2273773	−0.166 (0.278)	0.90	−0.155 (0.276)	0.38	0.443 (0.288)	0.12	0.03 (0.162)	0.28	−0.051 (0.17)	0.158 (0.291)	0.47
rs3740051	−0.22 (0.278)	0.58	−0.126 (0.277)	0.47	0.455 (0.288)	0.13	0.026 (0.162)	0.36	−0.103 (0.17)	0.206 (0.29)	0.28
***SREBF1***											
rs11656665	−0.317 (0.141)	0.011	−0.108 (0.149)	0.31	−0.087 (0.15)	0.08	−0.177 (0.085)	0.007	−0.139 (0.091)	−0.223 (0.148)	0.57
rs11868035	−0.335 (0.149)	0.003	−0.202 (0.160)	0.18	−0.2 (0.167)	0.10	−0.252 (0.091)	0.004	−0.147 (0.098)	−0.365 (0.161)	0.17
rs1889018	−0.422 (0.141)	0.001	−0.120 (0.149)	0.27	−0.09 (0.146)	0.06	−0.217 (0.084)	**0.002**	−0.189 (0.089)	−0.247 (0.147)	0.69
rs2236513	−0.443 (0.140)	0.001	−0.114 (0.150)	0.28	−0.069 (0.147)	0.09	−0.218 (0.084)	**0.003**	−0.159 (0.09)	−0.277 (0.147)	0.42
rs2297508	−0.398 (0.141)	0.001	−0.145 (0.150)	0.23	−0.071 (0.154)	0.12	−0.215 (0.086)	0.005	−0.136 (0.092)	−0.308 (0.15)	0.25
***TFAP2B***											
rs1569777	0.134 (0.189)	0.48	0.379 (0.231)	0.13	0.234 (0.222)	0.72	0.233 (0.122)	0.66	0.077 (0.133)	0.379 (0.211)	0.15
rs2076309	0.023 (0.138)	0.76	−0.185 (0.145)	0.20	−0.083 (0.145)	0.64	−0.079 (0.082)	0.34	−0.043 (0.088)	−0.132 (0.144)	0.54
rs2143079	0.363 (0.31)	0.13	−0.632 (0.307)	0.03	−0.445 (0.421)	0.11	−0.204 (0.193)	0.08	−0.088 (0.212)	−0.405 (0.334)	0.34
rs2206277	−0.087 (0.169)	0.93	0.409 (0.197)	0.11	0.093 (0.206)	0.54	0.115 (0.109)	0.28	0.082 (0.116)	0.212 (0.193)	0.50
rs2857506	−0.055 (0.213)	0.94	−0.207 (0.231)	0.38	−0.085 (0.236)	0.43	−0.113 (0.131)	0.64	−0.119 (0.139)	−0.098 (0.231)	0.93
rs7771651	0.008 (0.194)	0.97	−0.167 (0.205)	0.90	−0.061 (0.199)	0.76	−0.07 (0.115)	0.74	−0.018 (0.139)	−0.162 (0.203)	0.48
rs987237	−0.051 (0.172)	0.85	0.431 (0.197)	0.09	0.094 (0.206)	0.53	0.139 (0.11)	0.23	0.085 (0.116)	0.263 (0.195)	0.36

Effect estimates and standard errors (for the combined as well as sex-specific analyses) are based on the original adiponectin scale, whereas p-values are taken from the linear regression on log(adiponectin).

Recessive effect models as well as haplotype analyses did not provide any additional information (data not shown). There was also no significant sex-specific effect in regulation of adiponectin levels for SNPs in *FOXO1*, *SREBF1*, *TFAP2B* and *SIRT1* genes. Comparison of men-specific and women-specific data, however, showed a sex-difference in rs1801282 (*PPARG*, p for sex-difference  = 0.012), though this cannot be deemed significant given the number of tests involved. Nevertheless, it was interesting to see a negative effect in men (β = −0.235, p = 0.212), while the effect was positive in women (β = 0.288, p = 0.177).

## Discussion

Considering that transcriptional control of the *ADIPOQ* is one of the most important factors involved in regulation of adiponectin levels, we hypothesized that genetic variation at the loci encoding the main transcription factors controlling activity of the adiponectin promoter might be involved in regulation of adiponectin levels. We therefore performed association analyses of the 24 selected SNPs from 5 different transcription factors. We observed a modest influence of genetic polymorphisms at the *SREBF1* gene on the adiponectin levels in three healthy West-Eurasian populations including 8733 individuals, but not for the other transcription factors (*FOXO1, SIRT1, TFAP2B* and *PPARG)*. Two of the five investigated polymorphisms (rs1889018 and rs2236513) at the *SREBF1* gene locus demonstrated an influence on the adiponectin levels even after adjustment for multiple testing with lower concentrations in carriers of the minor allele. Additionally, our data revealed a sex-specific effect of the *Pro12Ala* SNP at the *PPARG* locus on adiponectin levels. The minor allele (*Ala*) of this gene negatively correlated with adiponectin levels in men, but positively in women. This finding was not significant after correction for multiple testing.

**Figure 2:**
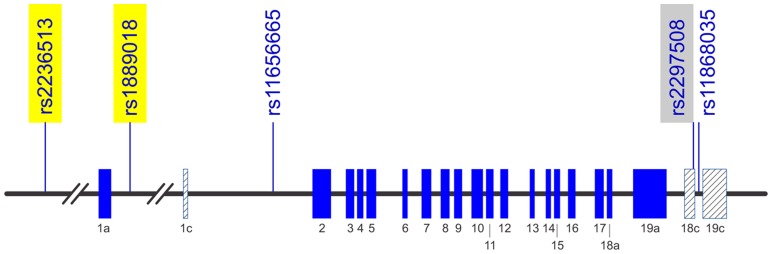
Schematic structure of *SREBF1* gene. Exons are numbered indicating the alternatively spliced -a and -c variants. Genomic location of the analyzed single nucleotide polymorphisms are marked. The single nucleotide polymorphisms highlighted in yellow showed a strongly associated with adiponectin levels in our study. The single nucleotide polymorphism highlighted in grey showed a significant association in a previous study [26] and was only borderline significantly associated in the present study (p = 0.004).

In previous publications the role of genetic polymorphisms of the transcription factors controlling *ADIPOQ* promoter activity in regulation of adiponectin levels was not investigated in detail – moreover the haplotype structure of these genes and sex-related effects (with the exception of *PPARG)* were not taken into consideration. Few years ago, Felder et al. using data from the SAPHIR cohort and additionally 446 unrelated patients with T2D discovered an association between one SNP at the *SREBF1* gene (rs2297508) and the prevalence of T2D and adiponectin levels [26]. In our study we extended the analysis of genetic polymorphisms at the *SREBF1* gene including additionally two cohorts and 4 SNPs, three of them having been selected as haplotype-tagging SNPs and one (rs2236513) based on literature data [40]. In our meta-analysis of 3 cohorts the two SNPs located in the 5'-UTR of the gene showed the strongest association with adiponectin levels (Figure 2). However, it should be noted, that all selected SNPs within *SREBF1* are highly correlated (Figure 1). Therefore, it can be assumed that the different hits within this gene refer to the same signal or signals. The search for the effect-triggering variants still requires further investigation. The identified SNPs at the *SREBF1* gene locus do not directly change its protein structure. They could, however, change various aspects of mRNA metabolism such as alterations of regulatory RNA-binding protein sites and mRNA secondary structure, that may influence functional properties of *SREBP1*c mRNA. It cannot be excluded that these SNPs are in linkage disequilibrium with yet unidentified functional mutations, either in the *SREBF1* gene or a gene located in that region.

The PPARG2 is a ligand-activated transcription factor, which acts as a heterodimer with the RXR [41], [42]. PPARG2 has been shown to increase transcription of the *ADIPOQ* gene and many other genes that are involved in the pathogenesis of insulin resistance [18], [19], [43]. Agonist-induced activation of PPARG is known to cause adipocyte differentiation, improvement in insulin sensitivity and also increased secretion of adiponectin by adipose tissue [44], [45]. The most frequently analysed and most well documented SNP in the *PPARG* gene is a proline to alanine substitution (*Pro12Ala*) in codon 12 of exon B (15% frequency among Caucasians). This substitution has a protective effect against the development of T2D. The *Ala* receptor variant is less efficient in its ability to bind and trans-activate a PPARG2 target gene in vitro [46]. The proline to alanine amino acid change also might affect the secondary structure of the protein and its functionality [47]. Heikkinen et al. (2009) suggested that the *Pro12Ala* polymorphism might be involved in the function of G protein, in sensitization of adiponectin signalling and altered recruitment of cofactors [48].

Yamamoto et al. investigated the effect of the *PPARG Pro12Ala* polymorphism on metabolic parameters and adiponectin levels in 598 Japanese people and found that adiponectin levels were significantly lower in subjects with the *Ala12* allele [33]. However, a Finnish study demonstrated that adiponectin levels were significantly higher among *PPARG*-*Ala* allele carriers after weight loss induced by heavy exercises [32]. In our study, we could not confirm an influence of the *Pro12Ala* polymorphism at the *PPARG* gene on adiponectin levels. Nevertheless, sex-specific effects of this SNP on adiponectin levels were observed indicating a positive correlation in women and a negative in men. The mechanism underlying this sex-specific effect of the *PPARG* gene in regulating adiponectin levels and various metabolic traits is currently not known. Sexual dimorphisms have frequently been reported in relation to fat distribution and have been evidenced for genes that affect BMI. Men are more likely to gain visceral fat and deep subcutaneous fat than women [49]–[51]. Taking into consideration that women have more subcutaneous fat as compared to men and PPARG2 expression is more pronounced in subcutaneous adipose tissue [52]–[54], one can speculate that sex-specific effects of this polymorphism on adiponectin levels are related to differences in fat distribution between men and women.

Our data did not reveal any effect of genetic polymorphisms at the *FOXO1*, *TFAP2B* and *SIRT1* gene loci on adiponectin levels. This might be explained by their minor role in the process of transcriptional regulation of the adiponectin gene or the investigated polymorphisms do not have sufficient influence on the structure of these transcription factor proteins.

Previous linkage studies suggested that different genetic components might be involved in the regulation of adiponectin levels, but their replications were inconsistent across different ethnic populations [6], [13], [15]–[17], [55]–[57]. Also the genetic variants detected in GWA studies did not explain the high level of heritability of adiponectin levels. The first GWA study was conducted in a European population and showed strong associations of adiponectin levels with *ADIPOQ* and *CDH13* loci [15]. Later, Richards et al. in a meta-analysis of three GWA studies confirmed *ADIPOQ* and revealed a new locus - *ARL15* (rs4311394) [58]. In 2010, Wu et al. provided a strong evidence of association with adiponectin for three loci: *ADIPOQ*, *CDH13* and *KNG1* together explained 7.5% and 8.9% of the variability of log-transformed adiponectin levels in Filipino women and their offspring, respectively. The strongest signal mapped to the *CDH13* and explained approximately 4% of the variability of adiponectin levels [59]. The association of *CDH13* locus with adiponectin levels was later confirmed in another GWA studies [14], [60], [61]. In a recent meta-analysis of GWA studies, 10 novel loci for adiponectin levels were identified and confirming the associations with variants at the *ADIPOQ* and *CDH13* loci [10]. The genes included in our study were not detected in the previously published GWA studies. Possible explanations for this finding might be the different principles of SNP selection (candidate gene approach in our study vs. use of common genetic determinants across the genome in GWA studies) and the much higher threshold for significance (p<10^−8^) used in GWA studies.

### Strengths and Limitations of the Study

The strength of our study is the selection of candidate genes based on the known regulatory mechanisms of adiponectin gene transcription. Additionally, we used the haplotype structure of the candidate genes for SNP selection with the addition of previously reported SNPs, allowing coverage of the whole gene region instead of single SNPs in the gene region. Finally, we could show direction-consistent significant effects for two SNPs in *SREBF1* gene in three European populations, including a total number of 8733 participants.

Nevertheless, several limitations of this work should be noted. The differences in mean age, proportion of male participants and subjects with T2D between the three populations studied might have influenced adiponectin levels [59], [62]. Also we did not take into consideration the genetic polymorphisms of all transcription factors known to be important for the adiponectin promoter, but included only four main transcription factors and one co-regulator based which have been found to be of greatest importance in regulation of adiponectin promoter activity and adiponectin levels. Finally, the study size was too small to performed analyses stratified for T2D or obesity.

### Conclusion

From the 24 selected SNPs at the five investigated transcription factors important for regulation of the adiponectin gene promoter, only those at the *SREBF1* gene had a modest influence on adiponectin levels in three healthy West-Eurasian populations. The role of genetic variations at the *SREBF1* gene and possible sex-related effects of *PPARG* in regulation of adiponectin levels have to be investigated in functional studies. Understanding the genetic mechanisms regulating adiponectin levels will expand our present knowledge concerning the factors that influence adiponectin levels. This could also lead to new therapeutic strategies to normalize circulating levels of adiponectin in subjects with metabolic disorders and cardiovascular disease.
